# Climate health: focus on translation

**DOI:** 10.1016/j.ebiom.2021.103782

**Published:** 2021-12-22

**Authors:** EBioMedicine

“Hope is often misunderstood. People tend to think that it is simply passive wishful thinking: I hope something will happen but I'm not going to do anything about it. This is indeed the opposite of real hope, which requires action and engagement.”

― Jane Goodall, *The Book of Hope* (2021)

With the release of The *Lancet*’s Countdown on Health and Climate Change in October, 2021, and the coincident UN Framework Convention on Climate Change 26th Conference of the Parties (COP26), sobering statistics across 44 indicators have made it clear that climate change is increasingly affecting our health—and that the situation is dire indeed.

As Dr Goodall describes in her new book, in order to have real hope in the midst of climate change, we have some work to do. As a translational research journal the overarching goal of our daily work is, simply put, to publish science that improves human health. What this means vis-à-vis climate health research is that we aim to better understand how climate and changing environments interface with our physiologies, and to identify therapies and interventions to counter negative effects due to our changing planet.

As outlined in “Hot weather and heat extremes: health risks” from The *Lancet*’s Heat and Health series published on Aug 21, 2021, one way in which our bodies deal with heat stress is by vasodilation, which helps transfer internal body heat to the external environment by increasing blood flow to the skin. This process requires increased cardiac output and oxygen demand. If prolonged and intense, the process can sometimes lead to serious cardiovascular events. As evidenced in a 2016 systematic review and meta-analyses published in *EBioMedicine*, the elderly face particularly high risk not only for heat-induced cardiovascular effects, but also negative cerebrovascular and respiratory outcomes.

Another way the body responds to heat is by sweating, whereby thermosensitive neurons in the hypothalamus instruct the sweat glands to release stored fluids and salts. This process, in turn, transfers heat energy away from the body via evaporation, resulting in a cooling of core body temperature. However, loss of fluid and subsequent dehydration can lead to further strain on the heart, as well as increase acute kidney injury and kidney failure in some vulnerable individuals. After prolonged heat exposure or exertion in hot temperatures, the body can lose its ability to thermoregulate and heatstroke can ensue. Heatstroke is a very serious condition that can lead to permanent damage to the brain, heart, and kidney, and even death. As with many health conditions, awareness of the dangers of heat exposure and the implementation of behavioural guidelines (such as taking breaks from exertion and staying hydrated during heatwaves) are effective methods for modifying some of these risks. However, research is urgently needed to better understand the pathophysiological mechanisms of heat dysregulation and its effects on the body, and to identify novel therapeutic targets and optimal cooling methods for patients in acute heat crisis.

Increased environmental heat causes plants to release more allergy-inducing pollen, and can create conditions that lead to unhealthy ozone levels. Dust and wildfires, brought on by intensified droughts due to climate change, can also release fine, inhalable particulate matter (PM_2•5_) that can infiltrate deep into the lungs and bloodstream. Fossil fuel combustion itself can directly contribute to fine particulate matter pollution and is attributable to an estimated 10•2 million premature deaths annually, according to a recent study in Environmental Research. These increases in allergens and pollutants intensify lung irritation, asthma-related symptoms, and have been linked to higher risk of lung cancer and death from cardiovascular complications. PM_2•5_ exposure has long been linked to severe outcomes during infectious disease outbreaks, and evidence is emerging that this association could also hold true for air pollution and COVID-19 disease severity.

The developing fetus is also susceptible to both excess heat exposure and air pollution, with each factor contributing to an increased risk for adverse outcomes, including preterm birth and low birthweight, as outlined in a systematic review published in JAMA Network Open. The effects of heat and pollutants on pregnancy are likely to involve complex and multifactorial mechanisms that are not yet well understood. However, some studies have suggested decreased uterine blood flow and increased proinflammatory cytokine release as possible contributing factors from heat stress, and direct fetal toxicity and inflammation among other factors mediating air pollutant effects. A recent study in Molecular Psychiatry, for example, found that prenatal exposure to PM_2•5_ might affect neurodevelopment and behaviour in autistic children via long-term effects on mitochondrial respiration.

Changes in temperature and environmental conditions can also contribute to increased incidence of infectious diseases by expanding the seasonal and geographic range of insect-vectored pathogens and by affecting migratory patterns of both people and insects. Research studies aimed at predicting spread of vector-borne illnesses, such as this 2016 *EBioMedicine* paper modelling Zika virus outbreak potential in Europe, are needed to inform public health risks of new climate-related epidemics.

At *EBioMedicine*, we put our hope in science to help us navigate a changing climate. We aim to publish work that helps us understand mechanisms of climate-related and environment-related health outcomes, and that provides new insights for climate-adaptive therapies or novel mitigations. We will consider research, review articles, and commentary all along the intersection of climate and human health, and encourage a cross-disciplinary collaborative research approach to help achieve our common goals.

